# Blood Serum Calorimetry Indicates the Chemotherapeutic Efficacy in Lung Cancer Treatment

**DOI:** 10.1038/s41598-017-17004-x

**Published:** 2017-12-01

**Authors:** Karolina Kędra-Królik, Izabela Chmielewska, Anna Michnik, Piotr Zarzycki

**Affiliations:** 1 Institute of Physical Chemistry, Polish Academy of Sciences, Kasprzaka 44/52, 01-224 Warsaw, Poland; 2 Department of Pneumonology, Oncology and Allergology Medical University of Lublin, Jaczewskiego 8, Lublin, 20-090 Poland; 3 Department of Medical Physics, A. Chełkowski Institute of Physics, Uniwersytecka 4, Katowice, 40-007 Poland; 4 Silesian Center for Education and Interdisciplinary Research, University of Silesia, 75th Pułku Piechoty 1A, Chorzów, 41-500 Poland; 5 Energy Geoscience Division, Lawrence Berkeley National Laboratory, 1 Cyclotron Road, Berkeley, CA 94720 United States

## Abstract

Chemotherapy is a primary treatment for the metastatic lung cancer patients. To select the most effective combination of drugs, we need an efficient way of assessing tumor response. Here, we showed that differential scanning calorimetry (DSC) analysis of blood serum proteins could reveal the patient response to the treatment. If chemotherapy is effective, serum proteins DSC curve of non-small cellular lung cancer (NSCLC) case is similar to the those of the healthy individuals. If treatment fails, notable changes occur in the DSC profile of NSCLC patient’s blood serum. Our preliminary work illustrates how thermal analysis of changes in the heat capacity of blood serum proteins can provide an insight into patient response to chemotherapy – the essential information for any successive lung cancer treatment.

## Introduction

Lung cancer remains the leading cause of cancer-related death worldwide^1,2^. The high mortality is due to late cancer diagnosis, at a stage when the tumor has already spread to other organs (metastasis)^3^. At this metastatic stage, treatment options are usually limited to chemotherapy or targeted therapy.

Almost all chemotherapeutic drugs have been designed to inhibit DNA replication or cell division in rapidly dividing tumor cells. However, these drugs efficacy is difficult to assess during the treatment.

At present, we rely on a frequent radiological scanning (e.g., spiral computed tomography) to determine therapy effectiveness by monitoring changes in tumors distribution and size^4^. On the other hand, the abundance of the blood samples makes it attractive to assess chemotherapeutic efficacy based on, for instance, the blood serum proteins. Unfortunately, it remains a challenge, mostly due to a lack of established lung cancer biomarkers.

Nonetheless, the blood serum proteins contain multiple cancer- and treatment-related signatures. First, the epigenetic changes associated with cancer manifest themselves in altered type, sequence and structure of expressed proteins at the cellular level (e.g., overexpression of growth factors and their receptors)^5,6^. Second, an effective chemotherapy reduces the number of cancerous cells, thus reducing the number of abnormally expressed proteins. Because the alteration of proteins is the cellular/extracellular signature of both malignant DNA alteration and chemotherapeutic drugs action, physicochemical analysis of cellular proteins should in principle allow one to detect treatment efficacy.

Among recently introduced methods for diagnostics and monitoring of diseases, the differential scanning calorimetry (DSC) seems to be the most promising one, regarding serum protein-based diagnosis^7–21^. DSC serum profile shows the heat capacity changes corresponding to the process of proteins unfolding as a function of a temperature. DSC evidences the absence or failure of protein structure or slight changes in protein stability. The weakness or stabilization of intermolecular interactions allows tracking of the molecules conformational change under the influence of environmental factors. The obtained DSC curve contains information about the thermodynamic stability of the most abundant serum proteins (i.e., their melting temperatures T_m_ and enthalpies of denaturation *ΔH*), protein mixture composition and the compositional changes taking place in the disease state, intra- and intermolecular interactions, including those related to alteration of protein structure. However, these data usually need skillful analysis because some of them are not directly available thus difficult to interpret. In applying DSC to blood serum proteins, one typically compares the denaturation curves obtained for a sick patient with the profile expected for the healthy individuals^7–17,20–22^. The disease-related alterations of serum proteins provide a diagnostic information evident on the DSC curve. DSC was tested to show the effects on blood serum caused by different injurious factors that are unavoidable in particular cases, e.g. necessary for treatment (chemotherapy, oxidative stress or neutron radiation)^18–21^. For instance, Ferencz *et al.* ^21^ showed that serum proteins denaturation profile of recovering breast cancer patients could indicate the tumor relapse. Here, we present the preliminary, but promising, results of DSC study of serum proteins obtained from metastatic lung cancer patients that respond positively or negatively to the platinum-based chemotherapy.

## Results and Discussion

**Figure 1:**
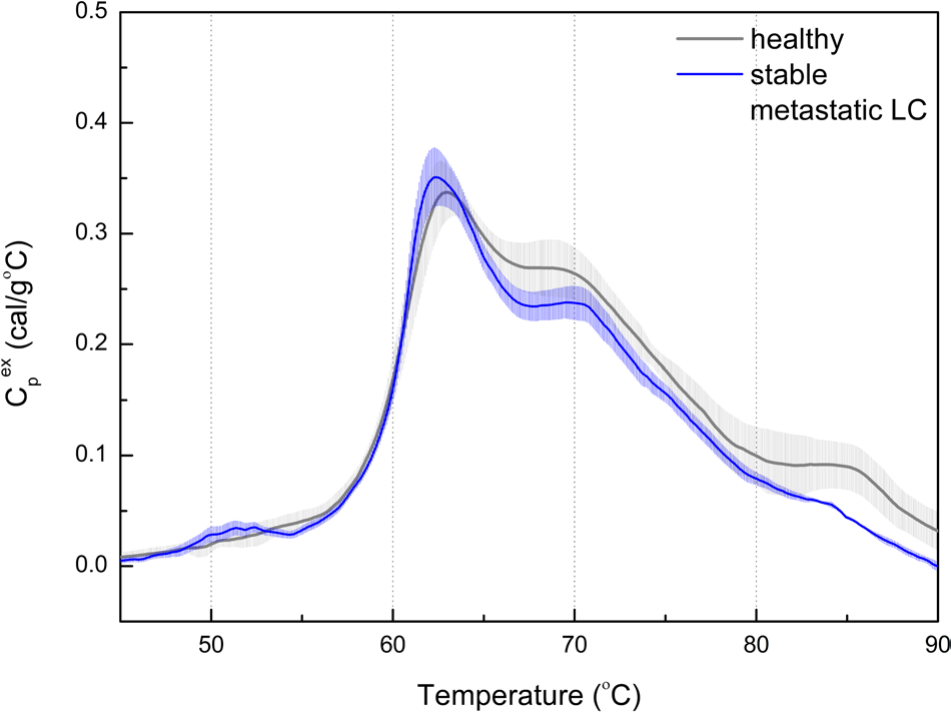
DSC curves obtained for blood serum samples from chemotherapy-treated LC patients with reduced progression (n = 8) and healthy individuals (n = 5).

**Figure 2:**
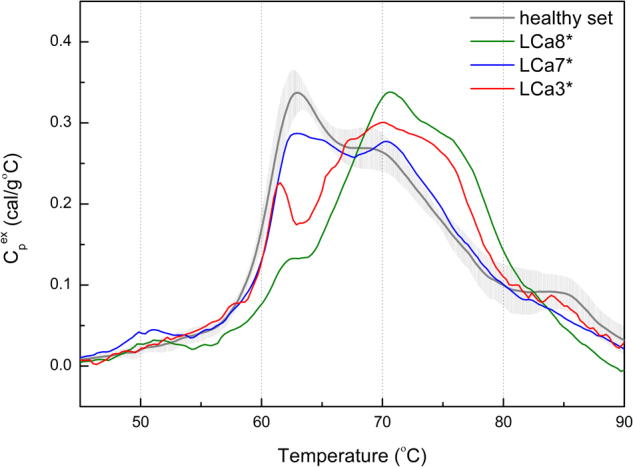
DSC curves obtained for blood serum samples from chemotherapy-treated LC with rapidly progressed adenocarcinoma patients (LCa7* without fluid in lungs; LCa8* and LCa3*- with massive fluid production); set with an average DSC profile for healthy individuals (n = 5).

For our preliminary study, we selected eleven non-small cellular lung cancer (NSCLC) patients (nine adenocarcinomas and two squamous cell lung cancer) at stage IV after administered platinum-based chemotherapy. The reference group of healthy individuals consists of two men and three women (mean age ~61). All samples groups were matched with ethnicity (West-Slavic group), age and smoking status. The lung-cancer patients were between 57 and 77 years old (mean value of 65.1), and five of them were smokers (see Table 1). Among investigated cases there was a group of three patients with pronounced genomic alterations. EGFR (epidermal growth factors receptor) mutation was detected using real-time PCR (polymerase chain reaction). ALK (anaplastic lymphoma kinase) rearrangement was confirmed based on immunohistochemistry and FISH (fluorescent *in situ* hybridization). Our DSC analysis of plasma samples yielded a normalized, baseline-corrected denaturation curve for each blood sample (see Figs 1 and 2). The DSC profile for our healthy volunteers agree well with the similar literature data^9^, and is a reference to the DSC curves of lung cancer patients. It is typically decomposed into four protein groups (i.e., peaks)^8,9^. The two dominant and most relevant for our study are: peak corresponding to unligated albumin with a trace of haptoglobin centered at about 63 C (T_m1_), and immunoglobulins peak located at about 70 C (T_m2_), with intensities Cp_1_^ex^, Cp_2_^ex^ respectively.

**Table 1:** Lung cancer patients - samples characteristics.

Sample (response to chemotherapy)	Total Proteins [mg·mL^−1^]	Albumin[%]	Sex	Smoking	Lung Cancer Type
LC-a1 (smLC)	75.0	58.7	M	Yes	Adenocarcinoma
LC-a2 (smLC)	75.0	58.5	F	No	Adenocarcinoma
LC-a3* (pLC)	67.0	57.2	F	No	Adenocarcinoma
LC-s4 (smLC)	68.0	58.5	F	No	Squamous cell carcinoma
LC-s5 (smLC)	65.0	62.5	M	Yes	Squamous cell carcinoma
LC-a6 (smLC)	78.0	55.6	F	No	Adenocarcinoma
LC-a7* (pLC)	65.0	60.1	F	No	Adenocarcinoma
LC-a8* (pLC)	76.0	56.6	F	No	Adenocarcinoma
LC-a9 (smLC)	66.0	64.5	F	Yes	Adenocarcinoma
LC-a10 (smLC)	64.0	62.5	M	Yes	Adenocarcinoma
LC-a11 (smLC)	62.0	62.3	M	Yes	Adenocarcinoma

A patient who responds to chemotherapy treatment is referred as smLC (stable metastatic lung cancer), whereas patient non-responding to the chemotherapy is referred as pLC* (progressive lung cancer).

The DSC profiles for all lung cancer patients (n = 11) differ from those of healthy ones (n = 5). The mean T_m1_ for the control group (63.00 ± 0.54) is slightly higher than for NSCLC patients (62.37 ± 0.77) but the difference is not statistically significant. However, the mean T_m2_ for NSCLC (69.85 ± 0.69) is higher than for the control group (68.86 ± 0.41) and the Student’s *t*-test shows statistically significant difference between these two groups (p = 0.01).

Eight out of eleven patients responded to chemotherapy, and they are referred as a stable metastatic lung cancer (smLC). Their DSC profiles resemble the one obtained for healthy individuals (see Fig. 1). It is not possible to show statistically significant differences between both subtypes of NSCLC, i.e., adenocarcinoma (mean age 65.6) and squamous cell lung cancer (mean age 62) due to the small sample size (only two patients with the second subtype). The smoking status doesn’t differentiate the shape of DSC curve.

In three patients the chemotherapy failed (referred as a progressive lung cancer, pLC*; mean age 65.3). The progressed patients had also EGFR mutation or ALK rearrangement. It is known that lung cancer patients with such abnormalities are more likely to respond to targeted treatment. Gross progression was the effect of ineffectual chemotherapy at moment of sampling. We observe that the serum DSC curve of chemotherapeutically non-responding patients (pLC*) differs significantly from those of the healthy or responding patients (Fig. 2). The results of a one – way ANOVA indicate statistically significant differences between these three groups in the Cp_1_^ex^ (p = 0.0003) as well as Cp_2_^ex^ intensities (p = 0.0006). The mean Cp_1_^ex^ value for pLC* patients (0.214 ± 0.077) is significantly lower than for smLC (0.387 ± 0.043; p = 0.001) and healthy (0.341 ± 0.023; p = 0.01) groups. The Cp_2_^ex^ value for pLC* patients (0.306 ± 0.031) is significantly higher than for smLC (0.230 ± 0.032; p = 0.02) and a little higher than for control group (0.270 ± 0.025). A similar change in a denaturation profile, i.e., a reduction of first denaturation peak and a significant increase in the high-temperature peak height, was reported by Garbett *et al* ^9^. for 30 lung cancer plasma samples. Another significant difference was found for T_m2_ parameter. It is higher for smLC and pLC* than for healthy group (p = 0.025 in Kruskal-Wallis test), particularly for pLC* patients (T_m2_ = 70.33 ± 0.22) in comparison with the healthy group (p = 0.02).

By analyzing clinical data, we confirm that chemotherapy was ineffective in this patients and we observed a massive tumor progression. Moreover, the DSC curves reveal the intensification of observed clinical symptoms of the developing disease. In two of progressing patients (LC-a3* and LC-a8*) rapid production and retention of fluid in lungs occurred. One can try to correlate the change in the DSC profile with the presence of EGFR activating mutation or ALK rearrangement. However, a chemotherapy-induced decrease in the albumin contents or alteration of interactions between serum proteins might also influence DSC curves^8,9^. The albumin fraction in chemotherapy responded patients is 55.6–64.5% of total proteins while for progressed patients 57.2–60.1%. It suggests that reduction of the first denaturation peak over 50% is not an effect of the quantitative albumin changes. It appears that a main fraction of the albumin peak shifts towards higher temperatures (with a maximum above 70 C, see Fig. 2). This indicates an increase in the thermodynamic stability of albumins as a result of modification of interactions in pathologically affected serum (i.e., biomarkers interactions with the major plasma proteins)^8,9^. Though our results are in agreement with the literature data, the unambiguous identification of the origin of altered DSC curve requires further investigation.

## Conclusions

We showed that the blood serum microcalorimetry is capable of differentiating metastatic lung cancer patients that respond well to chemotherapy from those who did not. If the selected chemotherapy drugs are effective, the DSC curve of serum proteins starts to resemble the DSC profile of the healthy individuals. What is more, a lack of tumor response to the treatment is also evident in the DSC curve. Even though serum protein DSC-based diagnosis is in its infancy and here we reported studies for a limited patient population, our results indicate that DSC can become a supportive method for monitoring lung cancer patient response to the chemotherapy.

## Methods

The informed consent from lung cancer patients and healthy volunteers was obtained before the peripheral blood samples were taken. The study was approved by Bioethics Committee at Medical University in Lublin and followed all local laws and regulations. The total serum proteins were determined using biuret method. The total albumin level was detected by UV-Vis spectrometry with bromocresol green.

The protocols used for the serum preparation included centrifugation and aliquoting before freezing. 5 ml Vacutainer tubes containing clot activator were used for blood collection. Samples were centrifuged 30 min after collection for 10 min at 10000 rpm. The supernatant was transferred into 2 mL Eppendorf microcentrifuge tubes and immediately frozen at −80 C to avoid proteins changes until the measurement. Straightway before the DSC analysis, the sample was thawed out at room temperature for 15 minutes. Then the 20-fold diluted solution was prepared using 0.25 ml of sample and 4.75 ml of degassed phosphate saline buffer (PBS) with pH 7.4 (0.1 M). Serum samples and reference solutions were degassed adequately before careful load into the cells to avoid bubble formation. The cell volume was 0.5137 ml.

The differential scanning calorimetry profiles were recorded using a VP DSC MicroCal micro-calorimeter (Northampton, MA) in the temperature range from 20 to 100 °C. The scan rate was 1 °C min^−1^ with a pre-scan equilibration time 15 min. The buffer–buffer scan under the same conditions was used as an instrumental baseline. For each sample, the temperature programs were performed two times. Serum DSC curves were normalized for the gram mass of protein and next linear baseline was subtracted. DSC curves represent changes of excess heat capacity, C_p_^ex^, (cal °C^−1^ g^−1^) in function of temperature.

The statistical analysis of parameters describing the thermal transition of serum (see data in the Table S1 in Supporting Information) was performed with Statistica 13. All results are given as mean values ± standard deviation. After checking for normal distribution (tested by Schapiro-Wilk test) and homogeneity of variance (Leven’s test), one-way analysis of variance (ANOVA) or independent- samples t-test for comparison of mean values were used. If ANOVA was statistically significant, Tukey’s post–hoc test for unequal sample sizes was applied. The Kruskal- Wallis H-test was used for non-normally distributed variables or in the case of non-homogenous variances. The level of statistical significance was set at p < 0.05.

### Ethical statement

Authors confirm that the project was approved by Bioethics Committee at Medical University in Lublin and followed all local laws and regulations. The informed consent from all volunteers was obtained before the peripheral blood samples were taken.

## Electronic supplementary material

Supplementary Information
