# Supplementary material for: Blood Serum Calorimetry Indicates the Chemotherapeutic Efficacy in Lung Cancer Treatment
Source: Sci Rep. 2017 Dec 1;7:16796. doi: 10.1038/s41598-017-17004-x (PMC5711924; doi:10.1038/s41598-017-17004-x)
Supplement: Supplementary file 1 — Supplementary Information [file 41598_2017_17004_MOESM1_ESM.pdf]

Supporting Information

Blood serum calorimetry indicates the chemotherapeutic efficacy in lung cancer treatment

Karolina Kędra-Królik[1,*] Izabela Chmielewska[2], Anna Michnik[3,4], and Piotr Zarzycki[5]

[1]Institute of Physical Chemistry, Polish Academy of Sciences, Kasprzaka 44/52, 01-224 Warsaw, Poland
[2]Department of Pneumonology, Oncology and Allergology, Medical University of Lublin, Jaczewskiego 8 Lublin, 20-090 Poland
[3]Department of Medical Physics, A. Chełkowski Institute of Physics, Uniwersytecka 4, Katowice, 40-007, Poland
[4]Silesian Center for Education and Interdisciplinary Research, University of Silesia, 75th Pułku Piechoty 1A, Chorzów, 41-500 Poland
[5]Energy Geoscience Division, Lawrence Berkeley National Laboratory, 1 Cyclotron Road Berkeley, CA 94720, United States
*kkedrakrolik@ichf.edu.pl

Table S1. Parameters of DSC curves for eleven lung cancer patients samples (LC-a – adenocarcinoma, LC-s – squamous cell lung cancer; * – progressing cancer) and healthy volunteers samples (H1-5). T_m – temperature at the peak maximum, Cp^{ex} – excess heat capacity at given T_m.

Sample	T_{m1} (°C)	Cp^{ex}_1 (cal g^{-1} C^{-1})	T_{m2} (°C)	Cp^{ex}_2 (cal g^{-1} C^{-1})
LC-a1	62.83	0.389	68.85	0.250
LC-a2	62.21	0.408	69.31	0.200
LC-a3*	61.49	0.221	70.09	0.303
LC-s4	61.51	0.456	69.16	0.170
LC-s5	62.58	0.411	68.82	0.221
LC-a6	63.40	0.367	70.07	0.247
LC-a7*	62.93	0.287	70.39	0.277
LC-a8*	62.79	0.133	70.52	0.338
LC-a9	63.36	0.324	70.02	0.259

LC-a10	61.58	0.337	70.37	0.230
LC-a11	61.42	0.405	70.75	0.263
H1	62.99	0.343	69.12	0.269
H2	63.23	0.345	68.56	0.308
H3	63.78	0.304	68.31	0.246
H4	62.42	0.345	69.08	0.277
H5	62.59	0.367	69.25	0.248
